# Calculous Phthisis

**Published:** 1855-02

**Authors:** 


### *Calculous Phthisis.*

(Translated for the Medical Examiner from the Gazette Medicale.)

M. Forget presented to the Academy of Medicine (meeting of 10th of Oct.,) a work entitled, Clinical view of Calculous Phthisis (not of tuberculous origin.)

From the facts stated by M. Forget, it should result:

1st. That pulmonary calculi can be primary, *sui generis*, that is to say, independent of the existence of tubercles, of inhaled dust, &c.

2d. These calculi can be solitary, either existing alone or in small numbers in the lungs.

3d. They may remain for a lesser or greater length of time, perhaps indefinitely, in a latent state in the lungs.

4th. They may occasion symptoms analogous to those of tuberculous phthisis.

5th. Calculous phthisis can be and is cured without relapse by expulsion of the pulmonary calculi, when they are either solitary or in small number.

6th. Hence calculous phthisis exists as a special affection, distinct from tubercular phthisis.

7th. Calculous phthisis differs essentially from tuberculous phthisis by its anatomical characters as well as by its terminations. Its differential diagnosis, drawn from its etiology, from its symptomatology, its course and treatment remain to be investigated.

